# Curing of Plasmid pXO1 from *Bacillus anthracis* Using Plasmid Incompatibility

**DOI:** 10.1371/journal.pone.0029875

**Published:** 2012-01-11

**Authors:** Xiankai Liu, Dongshu Wang, Huagui Wang, Erling Feng, Li Zhu, Hengliang Wang

**Affiliations:** State Key Laboratory of Pathogen and Biosecurity, Beijing Institute of Biotechnology, Beijing, China; Loyola University Medical Center, United States of America

## Abstract

The large plasmid pXO1 encoding the anthrax toxin is important for the virulence of *Bacillus anthracis*. It is essential to cure pXO1 from *B. anthracis* to evaluate its role in the pathogenesis of anthrax infection. Because conventional methods for curing plasmids (e.g., curing agents or growth at elevated temperatures) can induce mutations in the host chromosomal DNA, we developed a specific and reliable method to eliminate pXO1 from *B. anthracis* using plasmid incompatibility. Three putative replication origins of pXO1 were inserted into a temperature-sensitive plasmid to generate three incompatible plasmids. One of the three plasmids successfully eliminated the large plasmid pXO1 from *B. anthracis* vaccine strain A16R and wild type strain A16. These findings provided additional information about the replication/partitioning of pXO1 and demonstrated that introducing a small incompatible plasmid can generate plasmid-cured strains of *B. anthracis* without inducing spontaneous mutations in the host chromosome.

## Introduction

*Bacillus anthracis*, a gram-positive, rod-shaped, spore-forming bacterium, is the etiologic agent of anthrax, an acute and often fatal mammalian disease [1], [2], [3]. Virulent forms of *B. anthracis* harbor two large pathogenicity-related plasmids: pXO1 (181.6 kb), which encodes the anthrax toxin genes *pagA*, *lef*, and *cya* [2], [4]; the toxin regulatory elements *atxA* and *pagR*; the plasmid-borne germination genes *gerXC*, -*A*, and -*B* [2], [4], [5], [6]; and pXO2 (93.5 kb), which carries the genes responsible for capsule synthesis and degradation, *capA*, *-B*, *-C* and *-D* [7], [8], [9], [10], [11]. The two large plasmids of *B. anthracis* are essential for full pathogenicity; elimination of either dramatically attenuates the virulence of *B. anthracis*.

Numerous studies have attempted to characterize the role of these plasmids in virulence. Generating a plasmid-free strain is important to elucidate the crosstalk between the plasmids and host chromosome. Conventional curing strategies involve chemical agents (e.g., acridine orange, novobiocin, ethidium bromide), growth at elevated temperature, or treatment with ultraviolet (UV) light [12], [13], [14], [15], [16]. These strategies have been used successfully in many bacteria to eliminate resident plasmids. However, these methods are not specific to a particular plasmid but eliminate all resident plasmids. Furthermore, elimination of the resident plasmids is spontaneous; therefore, screening colonies for plasmid-cured strains is a tedious process. Finally, most known plasmid-curing chemical agents are toxic or mutagenic (e.g., acridine orange, ethidium bromide, and sodium dodecyl sulfate) and can therefore induce mutations in the host chromosome [17]. Mutations also occur during harsh physical curing treatments (e.g., high temperature or UV light). The phenotypic changes caused by mutation may interfere with the functional analysis of the plasmid [18]. For these reasons, there is a need to develop a curing method that is safe, reliable, and specific to the plasmid of interest.

Plasmid incompatibility is the inability of two different but related plasmids to coexist stably in the same host cell in the absence of continued selective pressure. Plasmids that share the same mechanisms for replication or partitioning are placed in the same incompatibility group. Plasmid incompatibility may be due to competition for the same replication or segregation sites, or caused by the repression of replication initiation [19]. Thus, introducing a smaller high-copy-number plasmid from the same incompatibility group may specifically eliminate a resident plasmid. This strategy has been demonstrated to be a specific, efficient, and safe technique to generate plasmid-free bacterial strains [20], [21].

Tinsley et al. isolated and characterized a minireplicon of plasmid pXO2 [22]. These researchers cloned a 2429-bp region of pXO2 into an *Escherichia coli* vector that is normally unable to replicate in *B. anthracis*, and demonstrated that this plasmid replicated when introduced into *B. anthracis*. The pXO2 replicon contains an open reading frame (ORF) encoding the putative replication initiation protein RepS and the putative origin of replication *ori*. Another ORF (*repB*) was included in this fragment but thought to be dispensable for replication of the pXO2 minireplicon [22]. Based on this information, we successfully cured the large pXO2 plasmid from the *B. anthracis* wild type strain A16 (pXO1^+^pXO2^+^) using a smaller plasmid in which a 3836-bp region of pXO2 containing *repS*, *repB*, and the putative pXO2 *ori* was inserted [23]. These results initiated our interest in eliminating the plasmid pXO1 from *B. anthracis* using plasmid incompatibility, which has not yet been reported in the literature. Little is known about the replication and partition properties of pXO1. The origin of replication and genes involved in replication initiation, plasmid partitioning, and plasmid stability have not been identified. Researchers have long sought to characterize the structure of the pXO1 replicon. Robertson et al. attempted to localize the replication origin by cloning pXO1 DNA fragments into an *E. coli* vector and transforming the plasmid into *B. subtilis*. This group identified several clones that mapped to an 11-kb region (coordinates 86249–97209) of the pXO1 sequence (GenBank accession no. AF065404; ORF72-81) (coordinate numbers and ORFs annotated by Okinaka et al. will be used throughout this report) [24]. The products encoded by the 10 ORFs did not show any sequence similarity to *rep* proteins associated with theta-replicating plasmids. In the past 10 years, data from numerous *B. anthracis* genome-sequencing projects have considerably increased our understanding of the biological roles of plasmids in this species. Plasmid pXO1 of the *B. anthracis* Sterne strain was sequenced by Okinaka et al. using a random whole-genome shotgun strategy, which enabled analysis of its replicon. The annotation predicted 143 ORFs in pXO1, but only 35 have putative functions assigned to them based on similarity to genes in open databases. Unfortunately, no sequences have been found that show significant similarity with known replication initiator proteins encoded by other plasmids [24]. Tinsley and Khan cloned a 5-kb region (ORF43–46, nt 54863–60166) of the plasmid pXO1 into an *E. coli* vector and demonstrated that this plasmid could replicate in *B. anthracis*. Mutational analysis showed that ORF45 was required for replication of the minireplicon, and this ORF was designated *repX* [25]. In a more recent effort to understand pXO1 replication, Andrei et al. isolated and characterized a new minireplicon of *B. anthracis* plasmid pXO1 using the Cre-loxP recombination system to delete large DNA segments; this region (ORF14–16, nt 19032–24981) differed from those previously described. Deletion analysis showed that only ORF14 and ORF16 were essential for replication of the minireplicon, and ORF16 was thought to be the replication initiator protein with a putative theta-replicating origin of replication [26].

In this report, we cured the virulence-associated plasmid pXO1 from *B. anthracis* vaccine strain A16R and wild type strain A16, using plasmid incompatibility to generate isogenic plasmid-cured and plasmid-containing strains. Our results will lead to a better understanding of the pXO1 plasmid and its interaction with the *B. anthracis* chromosome.

## Materials and Methods

### Bacterial Strains, Plasmids, and Culture

The bacterial strains and plasmids used in this study are listed in Table 1. *B. anthracis* A16 strain was isolated from the carcass of a mule that had died of anthrax in Langfang city in the Hebei province of China in 1953. The lethal dose of A16 is 100 spores for mice and 150 spores for rabbits administered by subcutaneous injection. *B. anthracis* vaccine strain A16R was derived from A16 by exposure to UV radiation. A culture dish containing the spore suspension of A16 was placed 10 cm from the center of a UV lamp. After a 3-hour UV treatment, the irradiated suspension was spread on agar plates containing 20% bovine serum and incubated in 20% CO_2_ at 37°C for 24 hours. A rough colony was chosen to streak on an agar plate containing 20% bovine serum, which was incubated in 20% CO_2_ at 37°C for 24 hours. This procedure was repeated until all descendants produced rough colonies. One of these clones was carried continuously in nutrient medium for 141 passages and in mice for 30 generations. With no descendants producing smooth colonies, this clone was considered stable and designated A16R. Its virulence was greatly attenuated, primarily through loss of the bacterial capsule, and A16R is now used for the human anthrax vaccine in China. For safety reasons and ease of handling in the laboratory, an attenuated *B. anthracis* vaccine strain A16R (pXO1+ pXO2−) was used for the initial experiments in the present study. The experiments were then repeated with the wild type strain A16. The temperature-sensitive shuttle vector pKSV7 (permissive temperature, 30°C; restrictive temperature, 37°C) contains a chloramphenicol resistance gene for selection in *B. anthracis*, an ampicillin resistance gene for selection in *E. coli*, and a multiple cloning site [27]. Strains containing recombinant plasmids were cultured at 30°C, except when curing the plasmid from the host bacteria, when the strains were cultured at 37°C. Before transformation into *B. anthracis*, all recombinant plasmids were passaged through *E. coli* SCS110 (*dam dcm* mutant) to demethylate the plasmid DNA, thereby facilitating its transformation into bacteria. All strains were cultured in Luria-Bertani (LB) broth (10 g/L tryptone [Oxoid, UK], 5 g/L yeast extract [Oxoid], 10 g/L NaCl) or on LB agar plates. Transformants were selected for ampicillin resistance (100 µg/mL) in *E. coli* or for chloramphenicol resistance (10 µg/mL) in *B. anthracis*.

**Table 1:** Plasmids and bacterial strains used in this study.

Plasmids and strains	Relevant genotype and characteristics	Source
pKSV7	Shuttle vector, temperature-sensitive, Amp^r^ (gram-negative) in *E. coli* and Cm^r^ (gram-positive) in *B. anthracis*	[27]
pKS4K	Incompatible plasmid, constructed by inserting 4-kb fragment of pXO1 into pKSV7	This work
pKS5K	Incompatible plasmid, constructed by inserting 5-kb fragment of pXO1 into pKSV7	This work
pKS11K	Incompatible plasmid, constructed by inserting 11-kb fragment of pXO1 into pKSV7	This work
*E. coli* DH5α	F^−^, ϕ80d/*lacZΔ*M15, *Δ*(*lacZYA*-argF)U169, *deoR*, *recA*1, *endA*1, *hsdR*17(r_k_^−^,m_k_^+^), *phoA*, *supE*44λ^−^, *thi*-1, *gyrA*96, *relA*1	This lab
*E. coli* SCS110	*rpsL*(StrR), *thr*, *leu*, *endA*, *thi*-1, *lacy*, *galK*, *galT*, *ara*, *tonA*, *tsx*, *dam*-, *dcm-*, *supE*44(lac-proAB), F- [traD36, proAB, lacIqlacZ*Δ*M15]	This lab
*B. anthracis* A16R	Vaccine strain, pXO1^+^, pXO2^−^	This lab
*B. anthracis* A16	Wild type A16R; pXO1^+^, pXO2^+^	This lab
*B. anthracis* A16R5K	A16R was cured of pXO1, harbors recombinant plasmid pKS5K; pXO1^−^, pXO2^−^, pKS5K^+^	This work
*B. anthracis* A16RO	pXO1 plasmid-cured derivative of vaccine strain A16R; pXO1^−^, pXO2^−^	This work
*B. anthracis* A16Q1	pXO1 plasmid-cured derivative of wild type A16; pXO1^−^, pXO2^+^	This work

### Construction of Incompatible Plasmids

**Figure 1:**
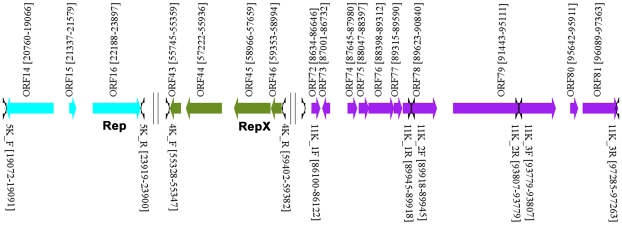
Three putative replicons of plasmid pXO1 (GenBank Accession no. AF065404). Each putative replicon of pXO1 is indicated in a different color, along with primer pairs used for amplification.

Based on the literature, we evaluated three DNA fragments (11-kb, 4-kb, and 5-kb) containing putative replication origins of pXO1 (Figure 1). The vector pKSV7 was linearized at the *SmaI* restriction site with restriction enzyme (TaKaRa Biotechnology, Dalian, China) and purified after 0.6% agarose gel electrophoresis using an agarose gel DNA purification kit. The 4-kb and 5-kb fragments were amplified with Pyrobest DNA polymerase (TaKaRa Biotechnology,Dalian, China) by polymerase chain reaction (PCR) from the total genomic DNA of strain A16R with the primer pairs 4K_F/R and 5K_F/R, respectively. The resulting fragments were cloned directly into the linearized pKSV7 vector by homologous recombination using the CloneEZ kit (GenScript, Piscataway, NJ, USA), generating the plasmids pKS4K and pKS5K, respectively. The 11-kb fragment was amplified by PCR from total DNA of strain A16R with the three primer pairs 11K_1F/R, 11K_2F/R, and 11K_3F/R. These PCR products were purified with the QIAquick PCR purification kit (Qiagen, Germany) and cloned in tandem into the linearized pKSV7 vector to generate pKS11K. PCR was performed in a 100-µL reaction containing 1 µL Pyrobest DNA polymerase, 10 µL 10× polymerase buffer, 8 µL dNTP mixture, 2 µL template DNA, 2 µL each primer (Table S1), and 75 µL double-distilled H_2_O (ddH_2_O). PCR amplification was carried out in a GeneAmp PCR System 9700 (Applied Biosystems, Carlsbad, CA, USA) with an initial denaturation step at 94°C for 5 min, followed by 30 cycles of 94°C for 40 s, 55°C to 58°C for 40 s, 72°C for 4 to 6 min, and a final extension step at 72°C for 5 min. Clones were generated by homologous recombination with the CloneEZ kit (GenScript) according to the manufacturer's instructions.

The plasmids pKS4K, pKS5K, and pKS11K were introduced into chemically competent *E. coli* DH5α cells. The samples were grown at 30°C for 3 h in fresh LB broth without antibiotics on a shaker (225 rpm) and then spread onto selective agar plates containing ampicillin. After incubation at 30°C for 12 to 16 h, transformants were picked and analyzed by colony PCR. Briefly, a single colony was suspended in 100 µL ddH_2_O, heated to 95°C for 2 min, and then cooled to room temperature. Cellular debris was removed by centrifugation at 15,000×*g* for 5 min, and 1 µL of the lysate was used as template for PCR amplification. PCR was performed in a 20-µL reaction containing 10 µL 2× Taq PCR Master Mix, 1 µL template, 7 µL ddH_2_O, and 1 µL each primer. The primer pair pKSV7-F/R, which flanks the multiple cloning site, was used to determine whether the 4-kb or 5-kb DNA fragment were inserted into pKSV7, and three primer pairs (11K_1F/R, 11K_2F/R and 11K_3F/R) were used to determine whether the 11-kb DNA fragment was inserted (Table S1). The thermal cycling conditions used to analyze transformants were same as those used to amplify fragments from the A16R total genomic DNA. Colonies that were positive for transformation, as assessed by colony PCR analysis, were analyzed by restriction digestion and DNA sequencing in an ABI Prism Model 3730XL DNA analyzer (Applied Biosystems). The recombinant plasmids pKS4K, pKS5K, and pKS11K were confirmed by sequencing and introduced into the *dam^−^ dcm^−^ E. coli* host strain SCS110 by electroporation (200 Ω, 25 µF, 1.8 kV) (Bio-Rad Gene Pulser II electroporator, Hercules, CA, USA) to obtain demethylated plasmid DNA for transformation into the *B. anthracis* A16R strain.

### Curing of Plasmid pXO1 from *B. anthracis* A16R Strain

The electroporation-competent *B. anthracis* A16R cells were prepared as previously described [28]. The plasmids pKS4K, pKS5K, and pKS11K were isolated (Axygen Scientific, Union City, CA, USA) from *E. coli* SCS110 and introduced into *B. anthracis* A16R by electroporation (500 Ω, 25 µF, 0.6 kV). The cells were grown at 30°C for 3 h in LB broth without antibiotics on a shaker (200 rpm) and then spread onto chloramphenicol-containing agar plates. After incubation at 30°C for 18 to 22 h, transformants were analyzed by colony PCR. Positive clones were selected and designated pKS4K/A16R (pXO1^+^pXO2^−^pKS4K^+^), pKS5K/A16R (pXO1^+^pXO2^−^pKS5K^+^), and pKS11K/A16R (pXO1^+^pXO2^−^pKS11K^+^), respectively. The three clones were passaged separately at least five times in 5 mL LB broth containing chloramphenicol (30°C, 225 rpm) for 12 h. At each passage, aliquots of each culture were diluted and spread on chloramphenicol-containing agar plates. Single clones were analyzed by colony PCR to determine the elimination of pXO1 from *B. anthracis* A16R using three pairs of primers (pag-F/R, lef-F/R, and cya-F/R) (Table S1). Putative pXO1-cured clones were selected, and genomic DNA was purified with TIANamp Bacteria DNA kit (Tiangen Biotech, Beijing, China) according to the manufacturer's instructions for isolating genomic DNA from gram-positive bacteria. To ensure the elimination of pXO1 from the A16R strain, 14 different pXO1 genes were analyzed by PCR using the total genomic DNA from strain A16R and the putative pXO1-cured clones as templates (Table S1).

### Elimination of Extraneous Plasmid pKS5K from Strain A16R5K (pXO1^−^pXO2^−^pKS5K)

To obtain a plasmid-cured strain without exogenous DNA, we needed to eliminate the recombinant plasmid from the host A16R strain. The pXO1-cured A16R strain containing the incompatible plasmids was passaged more than six times in 5 mL LB broth without antibiotics at 37°C for 12 h on a shaker (225 rpm). At each passage, aliquots of the cultures were diluted and spread on agar plates without antibiotics and incubated at 37°C for 12 h. Single clones were streaked onto two agar plates with or without chloramphenicol. The agar plates were incubated at 30°C overnight, and chloramphenicol-sensitive clones were considered positive for loss of the incompatible plasmid. The DNA of these clones was extracted and used as template for PCR with two primer pairs specific to plasmid pKSV7 (pKSV7P3_F/R and pKSV7P6_F/R, Table S1) to verify elimination of the incompatible plasmid.

### Detection of Anthrax Toxin Protective Antigen by Western Blot Analysis

Western blot analysis was performed to verify the curing of pXO1 from *B. anthracis* A16R. The A16R strain (pXO1^+^ pXO2^−^) and A16R derivative strain (pXO1^−^ pXO2^−^) were cultured on LB agar supplemented with 5% horse serum and 0.9% sodium bicarbonate at 37°C in 5% CO_2_ for 12 h. The bacteria were collected, and the cell suspension was separated by 12% bis-polyacrylamide gel electrophoresis. Proteins were transferred with Hoefer TE 77 semi-dry transfer unit (Amersham Bioscience, USA) from the gel to a polyvinylidene fluoride membrane with a constant current of 35 mA for 1 h 50 min. The transferred membrane was blocked with Tris buffered saline with 0.05% Tween 20 (TBST) containing 5% skim milk and then incubated with the mouse monoclonal antibody against protective antigen (C3; Santa Cruz, CA, USA) diluted in TBST (1∶500) for 60 min at room temperature. After washing three times with TBST for 7 min, the membrane was incubated in 40 ml TBST with goat anti-mouse IgG conjugated to horseradish peroxidase (HRP) (1∶10000) (Santa Cruz) for 60 min, and then washed four times with TBST for 7 min. The membrane was immersed in ECL substrate solution (Thermo Scientific, USA) for 5 min, and then exposed to x-ray film (Kodak RP X-OMAT, USA).

### Use of Plasmid Incompatibility to Cure pXO1 from Wild Type Strain A16

The plasmid used to cure pXO1 from *B. anthracis* vaccine strain A16 was introduced into *B. anthracis* wild type strain A16 to cure the plasmid pXO1 using the same methods described above. Bacterial capsules were stained with India ink (Gibco, USA) and observed with a phase-contrast microscope (Eclipse TE300, Nikon, Tokyo, Japan).

## Results

### Identification of Incompatible Plasmids

Colony PCR was performed to screen for transformants of the incompatible plasmids, and plasmids were isolated from the positive clones. Restriction analysis confirmed insertion of the 4-kb, 5-kb, and 11-kb DNA fragments into the vector pKSV7. The recombinant plasmids were confirmed by DNA sequencing and designated pKS4K, pKS5K, and pKS11K, respectively. These plasmids were introduced into *B. anthracis* to cure plasmid pXO1 from A16R.

### Elimination of pXO1 from A16R by Plasmid Incompatibility

**Figure 2:**
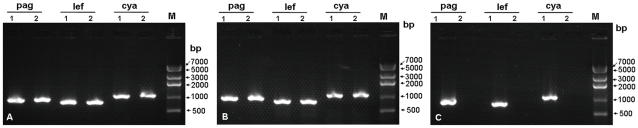
Colony PCR screening for pXO1 elimination with three plasmid-specific primer pairs. Results of using recombinant plasmids pKS11K (A), pKS4K (B), and pKS5K (C) to eliminate the large plasmid pXO1 from *B. anthracis* vaccine strain A16R by plasmid incompatibility. The presence of anthrax toxin genes *pagA*, *lef*, and *cya* was determined by PCR analysis of the vaccine strain A16R (1) and the putative pXO1-cured strain A16R (2). M, DNA marker IV (Real-Times Biotechnology, Beijing, China).

**Figure 3:**
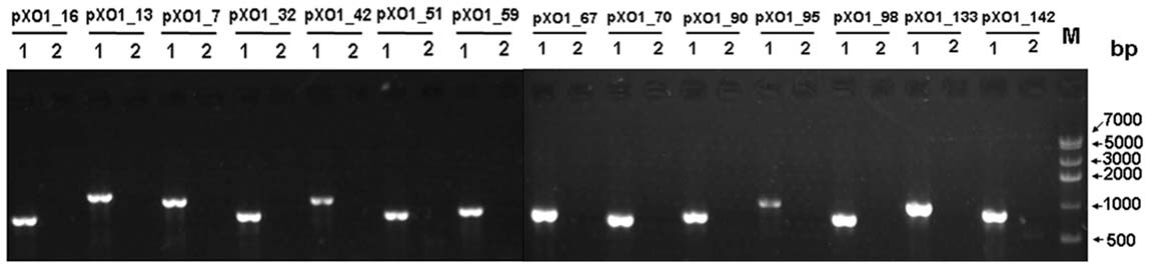
PCR analysis of pXO1 in *B. anthracis* vaccine strain A16R. PCR analysis of the vaccine strain A16R (1) and the putative pXO1-cured strain A16R (2) with 14 primer pairs specific for plasmid pXO1. M, DNA marker IV (Real-Times Biotechnology).

The recombinant plasmids pKS4K, pKS5K, and pKS11K were introduced into *B. anthracis* vaccine strain A16R and passaged 10 times in chloramphenicol-containing LB. The cultures were plated on selective LB agar plates containing chloramphenicol, and colony PCR was performed with the three pairs of specific primers (pag_F/R, lef_F/R, and cya_F/R) to screen for pXO1-cured colonies (Figure 2). Positive colonies underwent further PCR analysis using 14 pairs of specific primers to confirm the elimination of pXO1 from A16R. None of the primers specific to plasmid pXO1 amplified DNA from the A16R colonies transformed with pKS5K (Figure 3). These results demonstrate the elimination of pXO1 from *B. anthracis* A16R by the incompatible plasmid pKS5K. The pXO1-cured A16R derivative strain was designated A16R5K (pXO1^−^pXO2^−^pKS5K^+^). In contrast, the recombinant plasmids pKS4K and pKS11K did not eliminate pXO1 even after passaging the cells for more than 2 months in chloramphenicol-containing LB broth.

### Elimination of Exogenous Plasmid pKS5K from A16RK5 (pXO1^−^pXO2^−^pKS5K^+^)

**Figure 4:**
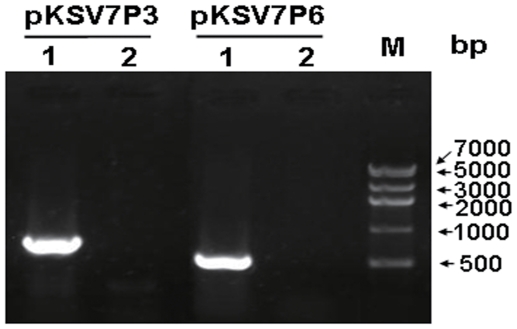
Elimination of exogenous plasmid pKS5K from A16R5K. Strains A16R5K (1), and A16RO (2) were analyzed with vector-specific primer pairs pKSV7P3_F/R and pKSV7P6_F/R. M, DNA marker IV (Real-Times Biotechnology).

After curing pXO1 from A16R, we needed to eliminate the exogenous plasmid pKS5K from the host bacteria. To avoid damage to the chromosomal DNA of A16R, the bacteria containing pKS5K were passaged five times in LB broth without antibiotic at 37°C. The culture was plated on LB agar without antibiotics, and single clones were screened by colony PCR with two primer pairs (pKSV7P3_F/R and pKSV7P6_F/R) specific to vector pKSV7. The pXO1-cured A16R derivative strain (A16R5K) and the candidate clones were analyzed by PCR to confirm elimination of the recombinant plasmid pKS5K. The predicted DNA fragments were amplified from DNA of A16R5K, but not from DNA of the candidate clones. These results indicate that the recombinant plasmid pKS5K was successfully eliminated from A16R5K, and this strain was named A16RO (pXO1^−^pXO2^−^) (Figure 4).

### Phenotypic Confirmation of A16RO (pXO1^−^pXO2^−^)

**Figure 5:**
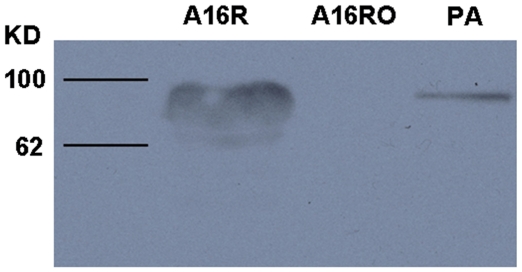
Western blot analysis of A16R and A16RO. The *B. anthracis* vaccine strain A16R (pX01^+^ pX02^−^) (left lane), A16RO (pX01^−^pX02^−^) (middle lane), and anthrax toxin protective antigen (PA) purified by affinity chromatography (right lane). The lines indicate the location of protein bands (Blue Plus Protein Marker, TransGen Biotechnology, Beijing, China).

To confirm elimination of pXO1 from strain A16RO, the presence or absence of the anthrax toxin protective antigen, encoded by pXO1, was determined by western blot analysis. The results revealed the presence of the protective antigen protein in the cell lysate of A16R, but this protein band was not detected in the cell lysate of A16RO, confirming that pXO1 had been eliminated (Figure 5).

### Use of Plasmid pKS5K to Cure pXO1 from Wild Type Strain A16 (pXO1^+^pXO2^+^)

**Figure 6:**
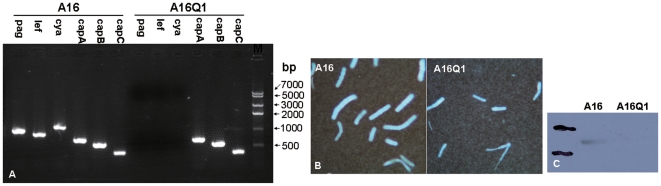
Use of plasmid pKS5K to cure the megaplasmid pXO1 from wild type strain A16. (A) Results of PCR analysis show the loss of anthrax toxin genes *pagA*, *lef*, and *cya* from pXO1-cured strain A16Q1. (B) Capsule formation was observed in both wild type strain A16 and pXO1-cured strain A16Q1. (C) Results of western blot analysis show the expression of anthrax toxin protective antigen protein in pA16, but not in A16Q1.

The results of PCR analysis and western blot analysis of the protective antigen demonstrated that pXO1 was successfully eliminated from *B. anthracis* wild type strain A16; this pXO1-cured strain was named A16Q1 (Figure 6). Although both strains produced capsules (Figure 6 B), the plasmid-cured strain A16Q1 did not produce the anthrax toxin protective antigen (Figure 6 C), which was consistent with the results of PCR analysis (Figure 6 A).

## Discussion

In this study, we aimed to eliminate the large plasmid pXO1 from *B. anthracis* using plasmid incompatibility. Although this strategy has been used in some bacterial species to cure resident plasmids, it has not yet been used in *B. anthracis* other than our previous work to eliminate plasmid pXO2 from *B. anthracis* wild type strain A16. This is mainly because the replication and partitioning of the virulence-associated plasmids pXO1 and pXO2 are poorly understood.

Characterizing the replication origin is important to eliminate resident plasmids from the host bacteria. However, only three studies have described the replication and partition properties of pXO1. Furthermore, the replication origins identified by the three reports differed; therefore, we tested the ability of three fragments, each containing one of the putative replication origins, to eliminate pXO1.

Three DNA fragments containing putative origin of replication of *B. anthracis* were inserted into a temperature-sensitive shuttle vector producing the curing recombinant plasmids. One fragment successfully eliminated pXO1 from vaccine strain *B. anthracis* A16R, and this result was repeated in wild type strain *B. anthracis* A16. Our results suggest that replication is not initiated at the putative pXO1 replication origins reported by Robertson et al. [24] and Tinsley et al. [25]. Robertson et al. investigated the replication origin of *B. anthracis* by transformation of a shuttle vector containing ORF72 to ORF80 of pXO1 into *B. subtilis*. However, replication of the shuttle vector in *B. subtilis* is not sufficient to infer its replication in *B. anthracis*. Tinsley et al. cloned several regions of plasmid pXO1 into the pBSCm plasmid of *E. coli* and then introduced these recombinant plasmids into *B. anthracis*. Because pBSCm is unable to replicate in *B. anthracis*, replication of the recombinant plasmids in *B. anthracis* suggested that the DNA fragments inserted into pBSCm contained the replication origin of pXO1. However, DNA regions that allow pBSCm to replicate in *B. anthracis* may not initiate replication of pXO1 in *B. anthracis*. After deleting these regions from pXO1, the plasmid should be tested in *B. anthracis* to determine whether it loses the ability to replicate.

Our results are in agreement with those of Andrei et al. [26] who characterized the pXO1 minimal replicon by DNA deletion. Andrei et al. reported that deleting a 5950-bp region of pXO1 prevented replication. We successfully used a 4848-bp DNA fragment within the 5950-bp region to cure pXO1 from *B. anthracis* using plasmid incompatibility. Our findings indicate that this fragment is the actual replication origin of plasmid pXO1.

In summary, we cured the plasmid pXO1 from *B. anthracis* A16R and A16 using plasmid incompatibility. This result has provided addition information about the replication and partitioning of pXO1. In addition, we developed a specific and reliable method to generate plasmid-cured strains of *B. anthracis* without inducing spontaneous mutations in the chromosomal DNA.

## Supporting Information

Table S1 Oligonucleotides primers used in this study.
(XLS)
